# Medication Misadventures Among COVID-19 Patients in Saudi Arabia

**DOI:** 10.7759/cureus.15513

**Published:** 2021-06-08

**Authors:** Dlal Almazrou, Oluwaseun Egunsola, Sheraz Ali, Amal Bagalb

**Affiliations:** 1 Pharmaceutical Care Services, King Saud Medical City, Ministry of Health, Riyadh, SAU; 2 Department of Community Health Sciences, University of Calgary, Calgary, CAN; 3 School of Pharmacy and Pharmacology, University of Tasmania, Hobart, AUS

**Keywords:** medication errors, adrs, covid, safety, adverse drug reactions

## Abstract

Background: Due to the need for early and effective medications for coronavirus disease (COVID-19), less attention may have been paid to medication safety during this pandemic.

Objectives: This study aimed to examine the incidence, nature, and seriousness of medication errors (MEs) and adverse drug reactions (ADRs) among hospitalized patients with COVID-19.

Materials and methods: This is a retrospective study of MEs and ADRs reported at the King Saud Medical City (KSMC) between April 2020 and September 2020.

Results: A total of 343 MEs and 416 ADRs were reported during the study period. The incidence of MEs was 19% (19/100). Seventy-five MEs (21.5%) reached the patient but did not cause any harm. Wrong dose (n=101, 29.4%) was the most common type of MEs. Physicians were the most common source of MEs (87.5%). Antibiotics (32%) and antineoplastics (25%) were the most common drug categories involved in MEs and ADRs, respectively. Thirty-nine percent (n=163) of the ADRs were of serious nature. 24% (n=100) required hospitalization, 5% (n=21) were life-threatening, 16 (3.8%) required intervention to prevent permanent impairment or damage, and 6.2% (n=26) resulted in the discontinuation of treatment.

Conclusion: The reporting of MEs appears to be high among COVID-19 patients in a large tertiary care setting in the Kingdom of Saudi Arabia (KSA). The majority of MEs were caused by dosing errors and errors in drug frequency, mostly ascribed to physicians, which may be indicative of burnout or stress among them. The reporting of MEs and ADRs can be improved by providing incentives to healthcare professionals (HCPs) and promoting a non-punitive culture. Further studies should explore the clinical consequences of medication misadventures in hospitalized COVID-19 patients.

## Introduction

Medication misadventure refers to “any iatrogenic hazard or incident associated with medications” [1]. It includes adverse drug events (ADEs) such as medication errors (MEs) and adverse drug reactions (ADRs) [1]. United States National Coordinating Council for Medication Error Reporting and Prevention (NCC-MERP) defines ME as “any preventable event that may cause or lead to inappropriate medication use or patient harm while the medication is in the control of the healthcare professional (HCPs), patient, or consumer. Such events may be related to professional practice, healthcare products, procedures, and systems, including prescribing, order communication, product labeling, packaging, and nomenclature, compounding, dispensing, distribution, administration, education, monitoring, and use” [2]. ADRs are defined as “a response to a drug which is noxious and unintended and which occurs at doses normally used in man for prophylaxis, diagnosis, or therapy of diseases or for modification of physiological function” [3]. MEs and ADRs are prevalent in hospitalized patients and can result in patient morbidity and mortality, and increased healthcare costs [4].

Approximately 130 million confirmed cases with about 2.8 million deaths have been linked to coronavirus disease (COVID-19) as of April 2, 2021 [5]. As of April 2, 2021, a total of 390,597 confirmed cases and 6,676 deaths were reported in the Kingdom of Saudi Arabia (KSA) [5]. Globally, there is no single proven effective therapy that is suitable for all mild, moderate, and severe cases of COVID-19 [6,7]. Although COVID-19 has no treatment, several drugs with different combinations are currently being investigated to treat COVID-19 [8]. Amid the COVID-19 crisis, patients in hospitals have been exposed to medications with unproven efficacy and safety for the prophylaxis and treatment of the disease, some of which may have compromised patient safety [9]. During this pandemic, pharmacists in the KSA have been integral to medication management in both clinical and non-clinical settings [10].

A report from China revealed that the incidence of medication-related problems in patients with COVID-19 was significantly high during the treatment period and 96.8% of medication-related problems occurred within 14 days [11]. In May 2020, the Institute for Safe Medication Practices in the United States also issued a warning about the emerging medication-related events linked to the COVID-19 pandemic [12]. Given the magnitude of unproven drug usage during COVID-19, it is crucial to ascertain its impact on medication misadventures such as MEs and ADRs. To the best of our knowledge, there is no published data on medication misadventures among hospitalized COVID-19 patients in the Arabian Gulf States. This study aims to fill the information gap by assessing the incidence, nature, and seriousness of MEs and ADRs in hospitalized COVID-19 patients in the KSA.

## Materials and methods

This study is a retrospective analysis of MEs and ADRs reported by HCPs at the King Saud Medical City (KSMC), the largest tertiary care setting in the KSA. It has 1,400 ward beds and about 140 intensive-care unit beds. All MEs and ADRs in COVID-19 patients reported between April 2020 and September 2020 were evaluated by pharmacists at the hospital’s Medication Safety Unit (MSU). The reporting of MEs and ADRs was a voluntary process in our study setting. MEs were directly reported to the MSU through an electronic ME report form. MSU pharmacists were responsible for routinely reviewing all the reported MEs and ADRs in the hospital. MSU pharmacists also reviewed patients’ medical records and contacted the reporter if any information required clarification. NCC MERP Index for categorizing MEs algorithm was used to determine the seriousness of the MEs [13].

In our study setting, ADRs were reported through an electronic report form. The MSU had access to the ADR reports submitted by the HCPs of KSMC. The seriousness of ADRs was determined according to the International Conference on Harmonization (ICH) E2A guideline [14]. According to the ICH E2A guideline, a serious ADR is any untoward medical occurrence at any dose that resulted in death, hospitalization or resulted in prolongation of existing hospitalization, persistent or significant disability/incapacity, congenital anomaly/birth defect, or medically important event or reaction that required medical/surgical intervention to prevent the serious outcome, and is life-threatening.

For this study, data for COVID-19 patients of any age were included. The following variables were included in this study: age, gender, type of MEs, stages of MEs, outcome of MEs, ADR, seriousness of ADR, and drugs involved in ADRs including dosage form and strength. The data were analyzed using the Statistical Package for the Social Sciences version 24 (IBM Corp., Armonk, NY, USA). Descriptive statistics were applied to the collected data. This study was initiated after the approval of the Institutional Review Board of KSMC (Reference number: H1RI-11-Aug20-03).

## Results

In total, 343 MEs were reported by HCPs between April 2020 and September 2020, for the 1,808 prescriptions ordered for COVID-19 patients during the study period. Thus, there were 19 MEs per 100 medication orders. The mean age of patients with reported MEs was 46.9 years (SD: 18.7) (Table 1). There were more males (n=246, 71.7%) than females. About 18% of MEs (n=62) involved high alert medications such as enoxaparin, morphine, fentanyl, and rocuronium, and 12% involved medications that looked alike or sounded alike (n=42). Physicians were the most common source of MEs (87.5%) (Table 1).

**Table 1:** Study demographics and characteristics in COVID-19 patients

Variables	Statistics
Age (years); mean ±SD	46.9 ±18.7
Age groups; n (n%)	
≤15 years	27 (7.9)
>15 years	316 (92.1)
Gender; n (n%)	
Male	246 (71.7)
Female	97 (28.3)
High Alert Medication; n (n%)	62 (18.1)
Look Alike Sound Alike Medication; n (n%)	42 (12.2)
Error Made by; n (n%)	
Physicians	300 (87.5)
Pharmacists	25 (7.3)
Pharmacy Technician	13 (3.8)
Nurses	5 (1.5)
Cause of Error	
Lack of staff education (competency validation, new or familiar drug/device, orientation process, feedback about errors).	145 (42.3)
Drug information missing (outdates/absent references, inadequate computer screening uncontrolled drug formulary).	91 (26.5)
Miscommunication of drug order (illegible, ambiguous, incomplete, misheard order, misunderstood order, and intimidation).	64 (18.7)
Clinical information missing (age, weight, allergy, vitals, lab, pregnancy, ID#, location, diagnosis, renal/liver impairment).	51 (14.9)
Environmental staffing or workflow problem (lighting, noise, clutter, interruption, staffing deficiency workload, employee safety).	50 (14.6)
Lack of quality control or independent check system (equipment quality control checks, independent checks for high alert medications/High risk patient population drugs).	35 (10.2)
Drug name, label, package problem (looks/sound-alike name, look alike packaging, unclear/no label, faulty drug identification).	17 (5)
Patient education problem (lack of information, on-compliance, not encourage to ask question, not investigating patient's inquiries).	12 (3.5)
Not supplied from Warehouses (unavailable medication)	9 (2.6)
Drug storage or delivery problem (slow turnaround time, inaccurate delivery, doses missing or expired, multiple concentrations, placed in the wrong bin).	6 (1.7)
Drug delivery device problem (poor device design, misprogramming, free flow, mixed up lines).	3 (0.9)

The most common causes of error were improper dose (n=101, 29.4%) and wrong frequency (n=84, 24.5%) (Table 2).

**Table 2:** Types of medication error in COVID-19 patients (n=343)

Medication Error	n (n%)
Improper dose (over, under or extra dose)	101 (29.4)
Wrong Frequency	84 (24.5)
Wrong Strength / Concentration	40 (11.7)
Wrong Drug	38 (11.1)
Omission Error	14 (4.1)
Wrong Duration	9 (2.6)
Wrong Route	5 (1.5)
Monitoring error-drug-drug interaction	3 (0.9)
Wrong Dosage Form	3 (0.9)
Wrong Patient	3 (0.9)
Monitoring error-drug-disease interaction	2 (0.6)
Monitoring error-clinical intervention	1 (0.3)
Wrong Rate of Infusion	1 (0.3)
Other	39 (11.4)

**Figure 1:**
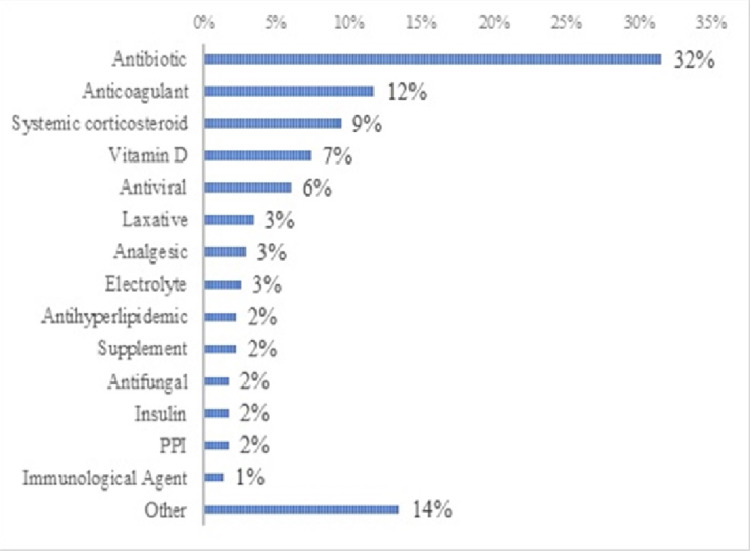
Most frequent drug category causing medication error (n=348)

Antibiotics and anticoagulants were the most common drugs with errors, constituting 32% and 12% of all errors, respectively (Figure 1). None of the MEs resulted in harm to the patients (Table 3).

**Table 3:** Percentage of medication errors in COVID-19 patients classified by the degree of patient harm according to NCC MERP ^a^Categories B-D were classified as no harm; categories E-I were classified as preventable adverse drug events NCC MERP = National Coordinating Council for Medication Error Reporting and Prevention

Classification	NCC MERP category ^a^	Definition	n	%
No error Error, no harm	A	Circumstances or events that have the capacity to cause error	134	39.1
	B	An error occurred, but the error did not reach the patient (near miss)	127	37
	C	An error occurred that reached the patient but did not cause patient harm	75	21.9
	D	An error occurred that reached the patient and required monitoring to confirm that it resulted in no harm to the patient and/or required intervention to preclude harm	7	2
Error, harm	E	An error occurred that may have contributed to or resulted in temporary harm to the patient and required intervention	0	0.00
	F	An error occurred that may have contributed to or resulted in temporary harm to the patient and required initial or prolonged hospitalization	0	0.00
	G	An error occurred that may have contributed to or resulted in permanent patient harm	0	0.00
	H	An error occurred that required intervention necessary to sustain life	0	0.00
Error, death	I	An error occurred that may have contributed to or resulted in the patient's death	0	0.00
Total			343	100

**Figure 2:**
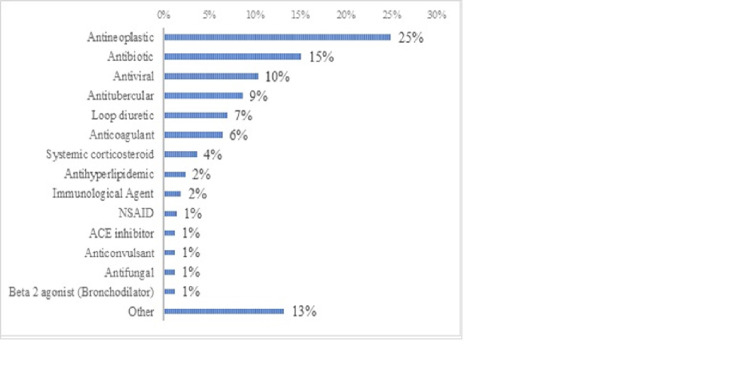
Most frequent drug category causing adverse drug reaction (n=416)

A total of 416 ADRs were reported during the study period. Antineoplastic drugs were the most common cause of ADRs, accounting for one-quarter of ADRs (Figure 2). Antibiotics and antivirals accounted for 15% and 10% of ADRs, respectively (Figure 2).

**Figure 3:**
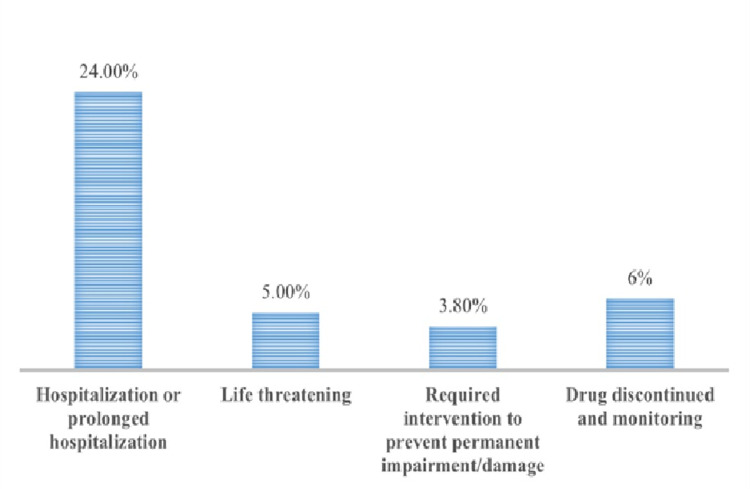
Percentage of the types of serious adverse reactions in COVID-19 patients (n=163)

One-hundred sixty-three of the 416 ADRs (39%) were serious, with 100 (24%) resulting in hospitalization, 21 (5%) life-threatening, 16 (3.8%) required intervention to prevent permanent impairment or damage, and 26 (6.2%) resulting in the discontinuation of treatment (Figure 3). 

## Discussion

This study shows that MEs and ADRs were frequently reported among COVID-19 patients at the KSMC hospital. The incidence of MEs among COVID-19 patients (19 MEs per 100 medication orders) was much higher than MEs reported during the pre-COVID-19 era (1.5 per 100 medication orders) at the same hospital [15]. However, the types of errors were similar during both periods. In the one 2015 pre-COVID-19 study, wrong frequency and wrong dose accounted for 27.8% and 21.1% of MEs, compared with 24.5% and 29.4%, respectively, in this study. MEs were unharmful to COVID-19 patients and to a large majority of the patients during the pre-COVID-19 period [15].

The higher incidence of reported MEs among COVID-19 patients may be ascribed to the increased stress, burnout, and anxiety among physicians, following exposure to COVID-19 patients [16,17]. However, this does not explain why physicians were the commonest source of errors, while nurses reported the fewest errors, given that both are patient-facing healthcare workers. Since physicians were responsible for most prescriptions, they would be the source of most errors. Several studies from our hospital found the pre-COVID-19 era MEs to be mostly transcribing errors [15,18], which typically involved nurses, who were required to transcribe prescriptions from the patients’ medical file onto the computerized order entry system. However, the hospital’s transition to direct prescription entry by physicians into the computerized order entry system [18] may have drastically minimized errors by nurses and consequently increased other types of physician errors. In one study, more than half of the physicians at a similar Saudi Arabian hospital indicated that migration to a computerized order entry system created a new type of error [19].

The rate of serious ADRs in this study was very high (39%), compared with 4% previously reported in the same hospital between 2015 and 2016 [20]. This may be due to the disproportionate reporting of serious ADRs among COVID-19 patients. The hospitalization rate following ADRs was also higher than the national rate of 2.1% reported in a 2018 national survey [21]. Furthermore, the profile of the drug classes causing ADRs was different from a pre-COVID-19 study [20]. Whereas anti-neoplastic drugs were not frequently associated with reported ADRs in the prior study [20], they constituted 25% of ADRs among COVID-19 patients in our study.

An important limitation of this study was the utilization of a voluntary reporting system, which has been associated with underreporting, caused by fear of retribution, lack of awareness about the available reporting methods, lack of time, or lack of interest and fear of disciplinary actions [22]. Voluntary reporting may not be a true representation of MEs in the hospital. Second, this study was conducted at a single tertiary hospital in KSA, thus the findings may not be generalizable for the country.

## Conclusions

In conclusion, the reporting of MEs appears to be high among COVID-19 patients in our hospital. The majority of MEs were caused by dosing errors and errors in drug frequency, mostly ascribed to physicians, which may be indicative of burnout or stress among them. The reporting of MEs and ADRs can be improved by providing incentives to HCPs and promoting a non-punitive culture. Further studies should explore the clinical consequences of medication misadventures in hospitalized COVID-19 patients.
